# Complex regulation of the regulator of synaptic plasticity histone deacetylase 2 in the rodent dorsal horn after peripheral injury

**DOI:** 10.1111/jnc.13621

**Published:** 2016-05-27

**Authors:** Maria Maiarù, Oakley B. Morgan, Keri K. Tochiki, Eleanor J. Hobbiger, Kaveeta Rajani, Dorothy W. U. Overington, Sandrine M. Géranton

**Affiliations:** ^1^ Cell and Developmental BiologyUniversity College LondonLondonUK

**Keywords:** astrocyte, Epigenetic, HDAC2, nitrosylation, nNOS, pain

## Abstract

Histone deacetylases (HDACs), HDAC2 in particular, have been shown to regulate various forms of learning and memory. Since cognitive processes share mechanisms with spinal nociceptive signalling, we decided to investigate the HDAC2 expression in the dorsal horn after peripheral injury. Using immunohistochemistry, we found that spinal HDAC2 was mainly seen in neurons and astrocytes, with neuronal expression in naïve tissue 2.6 times greater than that in astrocytes. Cysteine (S)‐nitrosylation of HDAC2 releases HDAC2 gene silencing and is controlled by nitric oxide (NO). A duration of 48 h after intraplantar injection of complete Freund's adjuvant, there was an ipsilateral increase in the most important NO‐producing enzyme in pain states, nitric oxide synthase (nNOS), accompanied by an increase in HDAC2 S‐nitrosylation. Moreover, a subset of nNOS‐positive neurons expressed cFos, a known target of HDAC2, suggesting that derepression of cFos expression following HDAC2 S‐nitrosylation might occur after noxious stimulation. We saw no change in global HDAC2 expression in both short‐ and long‐term pain states. However, HDAC2 was increased in astrocytes 7 days after neuropathic injury suggesting that HDAC2 might inhibit astrocytic gene expression in neuropathic pain states. All together, our results indicate that the epigenetic regulation of transcriptional programmes in the dorsal horn after injury is cell specific. Moreover, the prominent role of NO in persistent pain states suggests that HDAC2 S‐nitrosylation could play a crucial role in the regulation of gene expression leading to hypersensitivity.

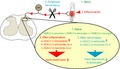

Our manuscript describes for the first time the regulation of the memory regulator histone deacetylase 2 (HDAC2) in the superficial dorsal horn of adult rats following peripheral injury. Our cell‐specific approach has revealed a complex pattern of expression of spinal HDAC2 that depends on the injury and the cell type, suggesting a sophisticated regulation of gene expression by HDAC2.

**Abbreviations used**
DAB: diaminobenzidinetetrahydrochloride
HDACs: histone deacetylases

## Background

The modulation of transcriptional programmes is a critical step for the plasticity mechanisms crucial to cognitive processes and also for nociceptive signalling (Géranton and Tochiki 2015a,b). It is achieved by alterations in chromatin compaction through changes in histone modification at the N‐terminal tail and is often accompanied by the recruitment of transcription factors to plasticity‐related genes. Histone deacetylases (HDACs) promote chromatin compaction and silencing of gene transcription by removing acetyl groups on histone tails. Another main function of HDACs is to maintain a low level of acetylation in primed genes to constrain their promoter in an inactive state but getting it ready for future rapid activation (Wang *et al*. 2009). HDACs have been shown to negatively regulate various forms of learning and memory (Penney and Tsai 2014). Furthermore, while synaptic plasticity and memory‐inducing paradigms promote histone acetylation in multiple brain regions, HDAC inhibitors improved cognitive performances in animal studies (Penney and Tsai 2014).

HDAC2, a class I HDAC, in particular, has been shown to play a central role in cognitive processes (Gräff *et al*. 2012; Penney and Tsai 2014). Deletion of HDAC2 in mice enhanced hippocampal plasticity and cognitive function indicating that HDAC2 negatively regulated memory formation (Guan *et al*. 2009). Crucially, HDAC2 binds the promoter and inhibits the acetylation of a number of plasticity‐related genes such as *BDNF, Fos* and *GluR1*. This process is modulated by cysteine nitrosylation (S‐nitrosylation), a signal particularly important in neurons where it can be regulated by the brain‐derived neurotrophic factor (BDNF) and neuronal nitric oxide synthase (nNOS) (Nott *et al*. 2008). While S‐nitrosylation of HDAC2 does not affect the deacetylase activity of HDAC2, it inhibits its association with its target genes, which leads to increased histone acetylation and possibly gene expression (Nott *et al*. 2008).

Levels of HDAC expression and histone acetylation have recently been the focus of a few studies in the context of spinal pain processing (Géranton and Tochiki 2015a,b). The reported changes are very complex and heterogeneous, but spinal level administration of inhibitors of class I HDACs (HDAC1, 2, 3 and 8) have temporarily reduced the hypersensitivity that develops after injection of complete Freund's adjuvant (CFA) in the hindpaw and nerve injury (Bai *et al*. 2010; Denk *et al*. 2013). These reports suggest that HDACs might play a role in the development and maintenance of long‐term pain states. HDAC2 is a key modulator of memory formation and therefore a likely regulator of nociceptive processing. Unfortunately, no HDAC2‐specific inhibitor exists, mainly because HDAC2 is structurally nearly identical to HDAC1 (Brunmeir *et al*. 2009; Haberland *et al*. 2009). Moreover, only small changes, if any, in spinal HDAC2 global levels have been reported following noxious stimulation (Bai *et al*. 2010; Tochiki *et al*. 2012; Géranton and Tochiki 2015a). Here, we have used a cell‐specific approach to investigate changes in HDAC2 expression in the superficial dorsal horn in a range of pain models and explored changes in spinal HDAC2 S‐nitrosylation.

## Methods

### Animals

Adult male Sprague–Dawley rats weighing 230–250 g at the time of surgery and obtained from the Biological Services Unit at University College London were used for all experiments. Rats were kept in their home cages in a temperature‐controlled (20 ± 1°C) environment, with a light–dark cycle of 12 h (light on at 7:30 a.m.), water and food were provided *ad libitum*. All efforts were made to minimise animal suffering and to reduce the number of animal used. All procedures were licensed under the United Kingdom Animals (Scientific Procedures) Act 1986.

### Animal models

#### CFA induced ankle joint inflammation

Inflammation was induced by injection of complete Freund's Adjuvant (CFA, Sigma, Dorset, UK; 10 μL) in the left ankle joint, under isoflurane anaesthesia induced in a chamber delivering 2% isoflurane combined with 100% O2 and maintained during injection *via* a face mask. The needle entered the ankle joint from the anterior and lateral posterior position, with the ankle kept in plantar flexion to open the joint. Sham treatment consisted of anesthetizing the animals. Sham animals are called ‘control’ animals throughout the manuscript.

#### CFA induced hindpaw inflammation

CFA (50 μL) was injected using a Hamilton syringe with a 27‐gauge needle subcutaneously into the plantar surface of the left hindpaw of rats. Rats were maintained under isoflurane anaesthesia during the injection. Sham treatment consisted of anesthetizing the animals. N.B.: sham animals did not receive any injection of vehicle. Sham animals are called ‘control’ animals throughout the manuscript.

#### Neuropathic model; spared nerve injury

The spared nerve injury (SNI) was performed according to Decosterd and Woolf (Decosterd and Woolf 2000). Briefly, under isoflurane anaesthesia, the skin on the lateral surface of the thigh was incised and a section made directly though the biceps femoris muscle exposing the sciatic nerve and its three terminal branches: the sural, the common peroneal and the tibial nerves. The common peroneal and tibial nerves were tight ligated with 5–0 silk and sectioned distal to the ligation. Great care was taken to avoid any contact with the spared sural nerve. Complete haemostasis was confirmed and the wound was sutured. Sham treatment consisted of exposing the sciatic nerve only.

### Western blot

Rats were deeply anesthetized with pentobarbital at 48 h post CFA injection. Spinal cord dorsal horn quadrants of the lumbar area (L4–L6) were dissected out, frozen on dry ice and kept at −80°C until further processing. For protein extraction, samples were each added to 250 μL lysis buffer (20 mM Tris‐HCl pH7.4, 150 mM NaCl, 1 mM EDTA, 1% Nonidet P‐40, in the presence of 1 mM phenylmethylsulfonyl fluoride and protease and phosphatase inhibitors). Samples were homogenized with a tissue disruptor (FastPrep FP120, ThermoSavant, GMI, Ramsey, Minnesota, USA), sonicated for 2 × 10 s, then left to incubate on ice for 30 min. Samples were centrifuged for 15 min, 14 000 rpm at +4°C, then supernatant was collected. Concentration of extracted protein lysates was determined using Bicinchoninic Acid protein assay (ThermoScientific). Samples were resolved on polyacrylamide gel (Biorad Criterion‐XT precast gel) and transferred onto polyvinylidene fluoride membrane (Bio‐Rad Laboratories, Hercules, CA, USA). After blocking for 1 h at 20–21 °C in 4% milk/0.1% phosphate‐buffered saline‐Tween20, the membranes were incubate with primary antibody (anti‐nNOS 1 : 2000, Cell Signalling, Danvers, MA, USA; Anti‐HDAC2 1 : 2000, Abcam, Cambridge, UK) O/N at 4°C. After several washes, an appropriate Horseradish Peroxidase‐conjugated secondary antibody was applied for 1 h at 20–21 °C. Blots were visualized with chemiluminescent enhanced chemiluminescence solution (Amersham Pharmacia Biotech, Piscataway, NJ, USA) or SuperSignal West Pico solution (Thermo Scientific) using BioRad ChemiDoc MP. Anti‐Calnexin antibody (1 : 1000, BioVision, San Francisco, CA, USA) was used as loading control. The membranes were visualized by BioRad ChemiDoc MP and signal intensity analysis was performed using ImageJ software (NIH, Bethesda, MD, USA). For quantitative analysis, the signal of each sample was normalized towards the corresponding Calnexin signal.

### Immunohistochemistry

For immunohistochemistry, rats were deeply anesthetized with pentobarbital and perfused transcardially with saline containing 5000 IU/mL heparin followed by 4% paraformaldehyde in 0.1 M phosphate buffer (PB). Lumbar spinal cords were dissected out, post‐fixed in the same paraformaldehyde solution for 2 h, and transferred into a 30% sucrose solution in PB containing 0.01% azide at +4°C, for a minimum of 24 h. Spinal cords were cut on a freezing microtome set at 40 μm. Sections were left to incubate with primary antibody O/N at 20–21 °C (anti‐HDAC2, 1 : 10 000 for Tyramide Signal Amplification (TSA) and 1 : 1000 for direct protocol, Abcam (ab32117), Cambridge, UK; or 1 : 5000 for TSA, Santa Cruz Biotechnology, sc‐7899, for Fig. S2 only; anti‐nNOS, 1 : 1000 for TSA, 1 : 2000 for diaminobenzidinetetrahydrochloride (DAB) peroxidase substrate and 1 : 500 for direct protocol, Cell Signalling; anti‐cFos, 1 : 1000, for TSA protocol Millipore, Darmstadt, Germany; anti‐NeuN 1 : 1000 for direct protocol, Millipore, Darmstadt, Germany; anti‐Glial Fibrillary Acidic Protein (GFAP) 1 : 4000 for direct protocol, DAKO, Cambridgeshire, UK; anti‐Iba1, 1 : 2000 for direct protocol, Wako, Osaka, Japan). For the direct protocol, direct secondary antibody was used at a concentration of 1 : 500 (Alexa Fluor, Thermo Fisher Scientific, Waltam, MA, USA). For TSA protocol, appropriate biotinylated secondary antibody was used at the concentration of 1 : 400 and left for 90 min. Sections were then incubated with avidin biotin complex (1 : 250 Vectastain A plus 1 : 250 Vectastain B; ABC Elite, Vector Lab, Peterborough, UK) for 30 min followed by a signal amplification step with biotinylated tyramide solution (1 : 75 for 7 min: Perkin Elmer, Wellesley, MA, USA). Finally, sections were incubated with FITC avidin for 2 h (1 : 600). To label nuclei, DAPI and TO‐PRO (Molecular Probes, Eugene, OR, USA) were used at concentrations recommended by the manufacturer. Sections were left to incubate for 10 min and 1 h, respectively, in PB solution at the end of the protocol. All fluorescent sections were coverslipped with Gel Mount Aqueous Mounting Medium (Sigma) to protect the fluorescence from fading and stored in dark boxes at +4°C. We ran controls when a double or triple stain was completed with two antibodies of the same host. A negative control eliminating the second primary always confirmed no cross‐reactivity of the secondary antibodies (Fig. S1). For the DAB protocol, sections were left to incubate with primary antibody overnight at 20–21 °C (anti‐nNOS, 1 : 2000, Cell Signalling) followed by conventional DAB protocol.

### Immunoprecipitation

Immunoprecipitation was carried out as described in Colussi *et al*. (2008). Briefly, samples were taken as described above and were each added to 250 μL immunoprecipitation buffer (20 mM Tris‐HCl pH7.4, 150 mM NaCl, 1 mM EDTA, 1% Nonidet P‐40, in the presence of 1 mM phenylmethylsulfonyl fluoride and protease and phosphatase inhibitors) and 10 lysing matrix‐D beads (MP Biomedicals, MP Biomedicals, Santa Ana, TX USA). A quantity of 75 μg protein lysates were incubated with 0.75 μL anti‐S‐nitrosylated cysteine (Cat#: NISC11‐A; Alpha Diagnostic, San Antonio, TX USA) antibody for 2 h at 20–21 °C. Magnetic protein‐A Bioadem‐beads (Ademtech, Pessac, France) were pre‐washed in the immunoprecipitation buffer described above; 20 μL of washed beads were added to each lysate sample and left to bind for another 2 h. Beads were washed several times before the protein was eluted in 13 μL PAG elution buffer (Ademtech). These samples were run on a western blot as described above and hybridized with anti‐HDAC2 antibody.

### Confocal imaging

All images of double and triple stained tissue were acquired by confocal microscopy using a laser‐scanning microscope (Leica TCS NT SP). Sequential laser channel acquisition was used to prevent the generation of false positives by ‘bleed through’ of immunofluorescence from one channel to the other. Images obtained were either single focal planes or Z‐stack series. For quantitative analysis of cell‐specific expression of HDAC2, a Z‐stack of four images taken across a 8 μm thick section (data presented in Fig. 2 and Fig. 3) or a Z‐stack of six images taken across a 10 μm thick section (data presented in Fig. 4) were acquired with an ACS APO 40.0 ×  oil objective. Images were always taken from paired ipsilateral and contralateral sides. For the analysis of HDAC2 expression in neurons and astrocytes (Fig. 2 and Fig. 3), a single picture per section side was taken spanning lamina I to III. For the analysis of HDAC2 expression in nNOS‐expressing neurons (Fig. 4), two pictures were taken: one covering the superficially expressed nNOS neurons in Lamina II and one covering the deeper nNOS‐positive neurons in Lamina 4. Offset and gain were fixed for sets from the same experiment. If in the process of acquisition, the intensity signal from some HDAC2 nuclei were saturated, the section was discarded from the analysis. A range of 4–6 dorsal horn sections were imaged per animal. Laser strength of each scan was not changed throughout the imaging.

### Fiji analysis

Fiji software (Schindelin *et al*. 2012) was used to quantify HDAC2 expression in the nuclei of neurons and astrocytes. To measure HDAC2 expression in neurons, the NeuN stain was used to create a mask of neuronal nuclei using fixed particle size and threshold across each study. HDAC2 immunohistochemical signal was measured within the NeuN mask. To measure the nNOS‐positive neurons, we created a mask of nNOS nuclei using the cytoplasmic stain obtained with the nNOS antibody, making sure to fill the hole because of the absence of nNOS stain in the nucleus. To measure the expression of HDAC2 in non‐nNOS‐positive neurons, the nNOS mask was subtracted from the NeuN mask. Since GFAP is not expressed within the nucleus of astrocytes, a very conservative method was followed to identify astrocytic nuclei. After excluding all neuronal nuclei from the image, only nuclei that were unequivocally concluded to be astrocytic after observation of the composite image of the Z‐stack were manually selected. Both for neuronal (including nNOS‐positive) and astrocytic HDAC2, HDAC2 intensity was recorded from a mask of HDAC2 nuclei created using fixed particle size and threshold across each study. The output measure from Fiji used for further analysis was the integrated density of the HDAC2 nuclei, which gave the most accurate representation of total HDAC2 expression per nucleus.

### Statistical analysis

All statistical analysis was performed using IBM SPSS Statistic 20. Data were analysed using repeated measures one‐ or two‐way anova, as relevant, and *post hoc* analysis was carried out with one‐way UNIVARIATE analysis.

## Results

### HDAC2 is mainly expressed in neurons and astrocytes in the superficial dorsal horn of naïve rats

**Figure 1:**
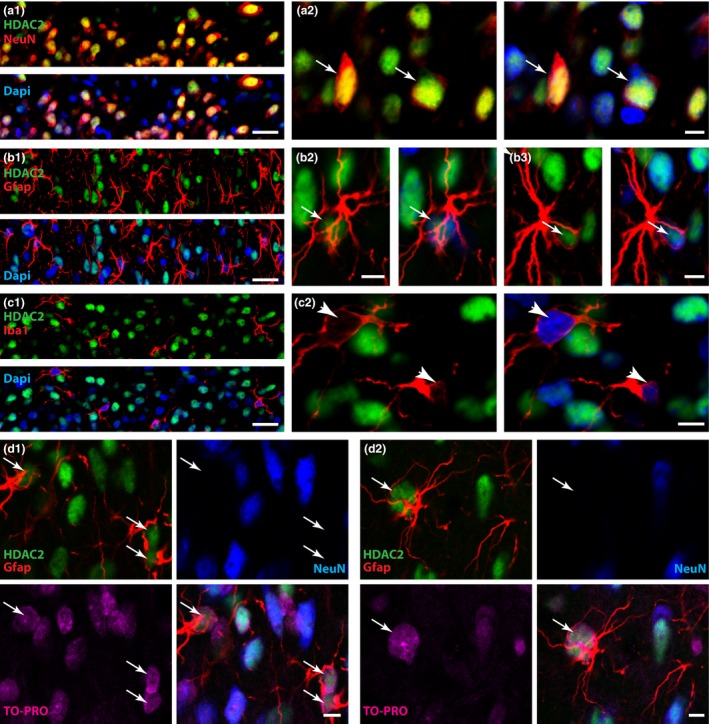
Rat spinal histone deacetylases (HDAC)2 is mainly found in neurons and astrocytes. HDAC2 expression in the rat superficial dorsal horn was investigated using immunohistochemistry. (a1–2) Expression of HDAC2 (green) in dorsal horn neurons (labelled with NeuN, red). Dapi (blue stain) was used to label nuclei. Coexistence is seen in yellow. All neurons were expressing HDAC2. (b1–3) Expression of HDAC2 (green) in dorsal horn astrocytes (labelled with Gfap, red). Since Gfap does not stain the astrocytic nucleus, there is no overlap between the two stains. Dapi (blue stain) was used to label nuclei. (c1–2) Expression of HDAC2 (green) in dorsal horn microglia (labelled with Iba1, red). Dapi (blue stain) was used to label nuclei. There was no obvious expression of HDAC2 in microglia. (d1–2) Expression of HDAC2 (green) in dorsal horn neurons (blue) and astrocytes (red). TO‐PRO (cyan stain) was used to label nuclei. Scale bar: a1, b1, c1: 20 μm; a2, b2, b3, c2, d1, d2: 5 μm. Arrows in A point at HDAC2 expressed in neuronal nuclei. Arrows in b and d point at HDAC2 expressed in astrocytic nuclei. Arrow head in C points at the absence of HDAC2 stain in microglial nuclei.

**Figure 2:**
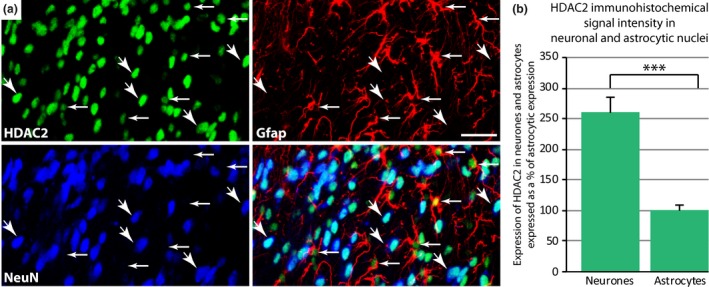
Rat spinal histone deacetylases (HDAC)2 expression is stronger in neurons than in astrocytes. (a) HDAC2 expression in neurons and astrocytes was investigated using immunohistochemical triple labelling. Horizontal arrows indicate HDAC2 expressed in astrocytic nuclei (astrocytes labelled in red) while diagonal arrows indicate HDAC2 expressed in neuronal nuclei (blue). (b) HDAC2 immunohistochemical signal intensity was measured in neurons and astrocytes. Quantification of HDAC2 expression using FIJI showed that HDAC2 expression was 2.6 times greater in neuronal nuclei than in astrocytic nuclei. Data normalized to astrocytic expression (100%). ***p < 0.01 result of Student's *t*‐test. Data show mean ± SEM. *N *= 8/8. Scale bar: 25 μm.

Using immunohistochemistry, we found strong expression of HDAC2 in dorsal horn neurons, labelled with NeuN (Fig. 1a, d1 and d2), and astrocytes, labelled with Gfap (Fig. 1b, d1 and d2). However, we could not detect any HDAC2 expression in dorsal horn microglia labelled with IBa1 (Fig. 1c). To confirm the validity of our findings, we repeated these stains with a different antibody (see Methods). Again, we found that HDAC2 was strongly expressed in neurons, to a lesser extent in astrocytes and was never seen in microglia (Fig. S2). We next quantified HDAC2 expression in neurons and astrocytes using triple labelling. We found that HDAC2 expression in the superficial dorsal horn of naïve rats was 2.6 times greater in neuronal nuclei than in astrocytic nuclei (Fig. 2).

### HDAC2 S‐nitrosylation increases in the dorsal horn 48 h following CFA injection in the hindpaw

**Figure 3:**
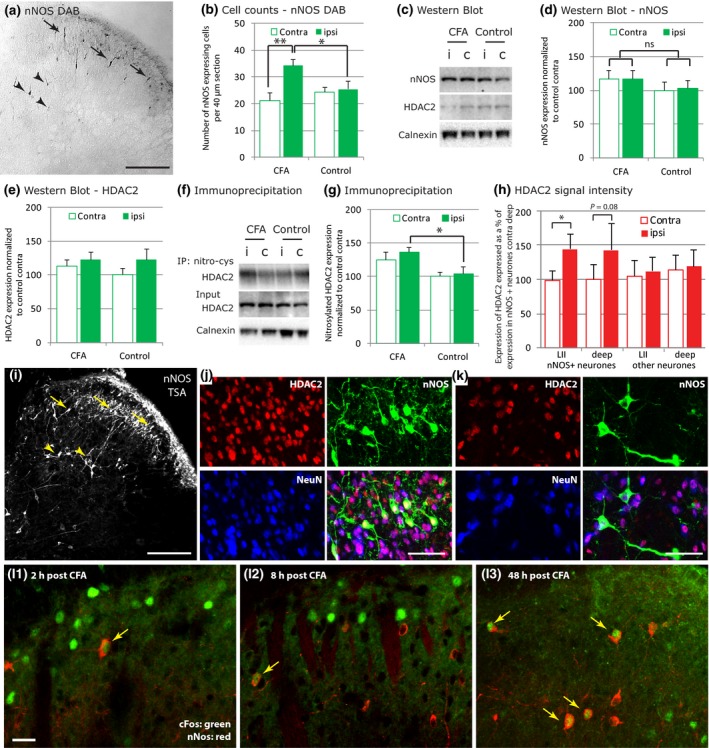
48 h after intraplantar CFA injection, spinal Histone deacetylases (HDAC)2 S‐nitrosylation increase. (a) Representative image of nNOS expression visualized using diaminobenzidinetetrahydrochloride (DAB) protocol in the rat dorsal horn. Note the nNOS‐positive neurons in lamina II (arrows) as well as the deeper neurons (arrow heads). Scale bar: 50 μm. (b) Using FIJI particle analysis we found an up‐regulation of the number of nNOS‐positive neurons per dorsal horn quadrant in the ipsilateral side of the injection. anova SIDE X TREAT:* F*_(1,8)_ = 10.4, *p* < 0.05; *Post hoc* analysis: CFA ipsi versus CFA contra: *F*_(1,6)_ = 33.1, *p* < 0.01; CFA ipsi versus control ipsi: *F*_(1,8)_ = 6.9, *p* < 0.05. *N* = 7(CFA)/3(control). (c and d) We found no significant increase in nNOS expression in the dorsal horn using western blot analysis. Data normalized to contra control (100%). *N* = 6/6. (c and e) Western blot analysis of HDAC2 expression indicated no changes 48 h following intraplantar CFA. Data normalized to contra control (100%). *N* = 6/6. (f) Representative blots after immunoprecipitation with a nitro‐cysteine antibody. (g) Quantification of immunoprecipitation blots. There was a significant increase in spinal HDAC2 nitrosylation 48 h after CFA injection. anova TREAT:* F*_(1,17)_ = 7.1, *p *< 0.05. *Post hoc* analysis for ipsi side: *F*_(1,18)_ = 5.9; *p* < 0.05. *N* = 10/10. (h) Using FIJI analysis, we found an up‐regulation of HDAC2 expression ipsi versus contra in nNOS‐positive neurons located in lamina II. Data normalized to nNOS‐positive neurons contra deep (100%). anova SIDE X HDAC2 EXPRESSION:* F*_(1,12)_ = 4.6, *p *< 0.05. *Post hoc* analysis paired *t*‐test. *N* = 4. (i) nNOS expression observed after immunohistochemistry following the TSA amplification protocol. Note the nNOS positive neurons in the superficial lamina II (arrows) as well as the deeper neurons (arrow heads). Scale bar: 200 μm. (j–k) Representative images of nNOS (green), HDAC2 (red) and NeuN (blue) expression in (j), lamina II and (k), lamina IV‐V. Scale bar: 50 μm. (l1–3) cFos (green) and nNOS (red) positive neurons in the superficial dorsal horn, 2, 8 and 48 h post CFA injection in the hindpaw. Arrows point at nNOS nuclei positively labelled with cFos. Scale bar: 25 μm. (b, d, e, g and h) Data show mean ± SEM. **p < 0.01; *p < 0.1. I, ipsi; C: contra; IP: immunoprecipitation.

We then explored the possibility that spinal HDAC2 underwent S–nitrosylation following noxious stimulation. S‐nitrosylation is controlled by nitric oxide (NO) and nNOS is the most important NO‐producing enzyme in the spinal cord during the development and maintenance of persistent pain states (Tao *et al*. 2004; Chu *et al*. 2005; Boettger *et al*. 2007; Guan *et al*. 2007). Increased expression of nNOS had been reported in the dorsal horn within 48 h of noxious stimulation (Herdegen *et al*. 1994; Yonehara *et al*. 1997; Maihöfner *et al*. 2000; Chu *et al*. 2005). We found a significant increase in the number of nNOS‐positive cells on the ipsilateral side following intraplantar CFA injection using DAB immunostaining technique (Fig. 3a and b), but we did not find any significant increase in nNOS levels using western blot analysis (Fig. 3c and d). Moreover, we did not measure any difference in HDAC2 expression in the dorsal horn using western blot analysis (Fig. 3c and e). However, we investigated changes in HDAC2 S–nitrosylation using immunoprecipitation and found that 48 h following CFA injection in the ankle joint, there was a significant global increase in HDAC2 S–nitrosylation in the ipsilateral dorsal horn (Fig. 3f and g). Using immunohistochemistry and a cell specific analysis, we found that HDAC2 expression was significantly up‐regulated 48 h following intraplantar CFA in nNOS+ neurons in lamina II (Fig. 3h, i, j and k). However, there was no change in HDAC2 expression in other neuronal cells analysed as a single group. Finally, HDAC2 has been shown to regulate the expression of the immediate early gene *Fos* (Penney and Tsai 2014) and a subset of nNOS‐positive neurons have been shown to express cFos 2 h following formalin injection (Polgár *et al*. 2013). We therefore explored the possibility that HDAC2 regulation of cFos expression could occur in nNOS‐positive neurons. Two hours and eight hours following CFA injection in the hindpaw, we never saw more than 1 nNOS‐positive cell expressing cFos per 40 μm section and there was no difference in the number of nNOS‐expressing cells between the ipsilateral and contralateral side (average of 27.1 nuclei on the ipsilateral side and 26.9 nuclei on the contralateral side; 23.6 nuclei on the ipsilateral side and 21.6 nuclei on the contralateral side, at 2 h and 8 h respectively; *n* = 3/3; Fig. L1, 2). However, 48 h following CFA injection in the hindpaw, 7% of nNOS‐positive neurons expressed cFos (8.5% of cFos‐expressing neurons expressed nNOS; average of 18.2 cFos nuclei on the ipsilateral side and 6.4 nuclei on the contralateral side; *n* = 2; Fig. L3).

### There is no global change in neuronal HDAC2 expression in the dorsal horn 7 days after CFA injection in the ankle joint or 7 days following neuropathic injury

**Figure 4:**
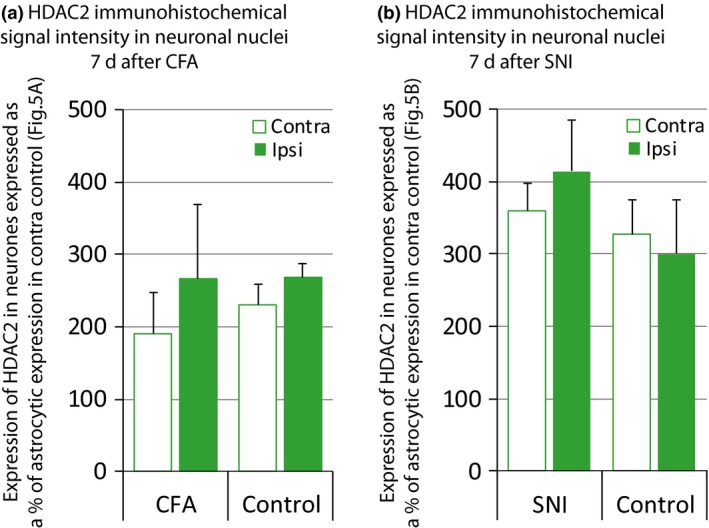
There is no change in global neuronal histone deacetylases (HDAC)2 expression in long‐term pain states. HDAC2 expression in neurons was investigated using immunohistochemical triple labelling as in Fig.2. HDAC2 immunohistochemical signal intensity was quantified using FIJI. (a) 7 days following ankle joint inflammation, neuronal HDAC2 expression remained unchanged. Data normalized to astrocytes contra control (100%; Fig. 5a). *N* = 4/4. (b) 7 days post spared nerve injury (SNI) surgery, neuronal HDAC2 expression was the same as in control treated animals. Data normalized to astrocytes contra control (100%; Fig. 5b). *N* = 4/4. Data show mean ± SEM.

We used immunohistochemistry to investigate HDAC2 protein expression specifically in neurons, all subtypes confounded, of the superficial dorsal horn in long‐term pain states. We found no global changes in HDAC2 expression in neurons 7 days after CFA injection in the ankle joint (Fig. 4a) or 7 days following SNI surgery (Fig. 4b).

### Spinal HDAC2 expression changes in astrocytes after nerve injury but not after ankle joint inflammation

**Figure 5:**
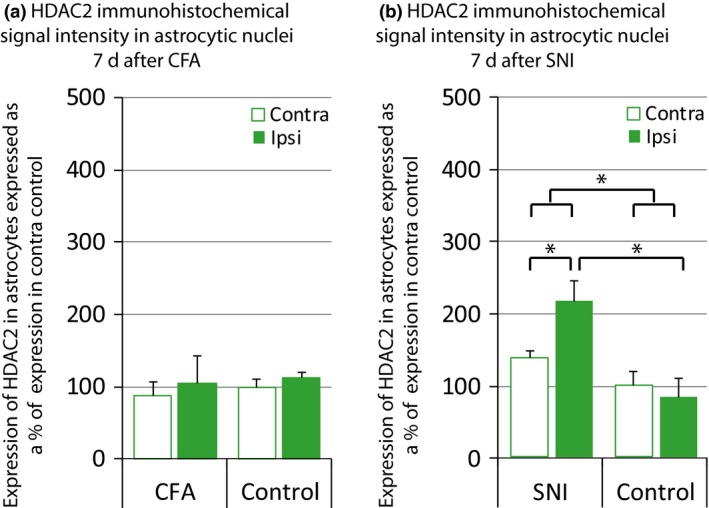
Spinal histone deacetylases (HDAC) 2 expression changes in astrocytes after nerve injury, but not after ankle joint inflammation. HDAC2 expression in neurons was investigated using immunohistochemical triple labelling as in Fig. 2. HDAC2 immunohistochemical signal intensity was quantified using FIJI. (a) 7 days following ankle joint inflammation, astrocytic HDAC2 expression remained unchanged. Data normalized to astrocytes contra control (100%). *N* = 4/4. (b) There was a significant increase in HDAC2 expression in astrocytes when compared with control, 7 days post spared nerve injury (SNI) surgery. Data normalized to astrocytes contra control (100%). *N *= 4/4. anova TREAT *F*_(1,5)_ = 8.5, *p *< 0.05; *Post hoc* analysis: for SNI only: *F*_(1,3)_ = 14.4, *p* < 0.05 and SNI ipsi versus control ipsi: *F*_(1,5)_ = 10.5, *p* < 0.05. *p < 0.05. Data show mean ± SEM.

We finally explored whether astrocytic expression of HDAC2 might change in long‐term pain states. We found no changes in HDAC2 expression in astrocytes 7 days after CFA injection in the ankle joint (Fig. 5a), but we found that HDAC2 expression was increased in spinal astrocytes 7 days following SNI surgery (Fig. 5b).

## Discussion

While the inhibitory role of HDAC2 in cognitive processes such as hippocampal‐dependent memory formation has been acknowledged for some time, its role in the plasticity observed in the spinal nociceptive pathways following injury has never been investigated. Here, we report that HDAC2 was clearly expressed in both neurons and astrocytes, with a greater expression level in neurons. There was a global increase in HDAC2 S‐nitrosylation in the dorsal horn 48 h post‐CFA injection suggesting that HDAC2 repression of gene expression was likely to be released. Global expression of HDAC2 did not change in short‐term or long‐term pain states, but HDAC2 expression in nNOS‐positive cells and in astrocytes increased 48 h after CFA and 7 days after SNI surgery respectively. This suggested that regulation of gene expression by spinal HDAC2 was cell specific and highly complex.

First, we investigated the cell‐specific expression of HDAC2 in the rat superficial dorsal horn. HDAC2 is generally thought to be ubiquitously expressed and we found that HDAC2 was strongly expressed in spinal neurons and to a lesser extent in spinal astrocytes. However, we never saw any HDAC2 signal in microglial cells. This was somewhat surprising as it has been shown that HDAC2 mRNA is present in cortical microglia (Gosselin *et al*. 2014; Lavin *et al*. 2014). However, our findings support data published by others indicating that throughout the adult mouse brain, HDAC2 was never observed in microglia (Yao *et al*. 2013), which suggested that cortical HDAC2 mRNA might never be translated. Another study in the murine brain also indicated that while HDAC2 is initiated in neural progenitors and up‐regulated in post‐mitotic neuroblasts and neurons, it is not expressed in fully differentiated glia (MacDonald and Roskams 2008). Interestingly, these studies did not detect HDAC2 expression in astrocytes in adult murine brain. The protocol of amplification used, however, was not as effective as ours (TSA protocol), which could explain why they did not detect lower expression levels of HDAC2. Another reason could be that HDAC2 cell‐specific expression in the rodent brain differs from that in the spinal cord.

We then decided to investigate whether HDCA2 could be S‐nitrosylated following peripheral noxious stimulation. S‐nitrosylation is controlled by nitric oxide (NO), which is produced at spinal level after noxious stimulation, and occurs at Cys262 and Cys274 on HDAC2, promoting transcription following HDAC2 dissociation from the DNA. nNOS is the most important NO‐producing enzyme in the spinal cord during the development and maintenance of persistent pain states (Tao *et al*. 2004; Chu *et al*. 2005; Boettger *et al*. 2007; Guan *et al*. 2007). nNOS is expressed in the naïve rat superficial dorsal horn and an increase in the number of spinal nNOS‐positive neurons during the development of inflammatory pain states has been reported by a number of studies (Herdegen *et al*. 1994; Yonehara *et al*. 1997; Maihöfner *et al*. 2000; Chu *et al*. 2005). Our experiments suggested that the greatest up‐regulation of nNOS‐positive cells in the superficial dorsal horn early after CFA injection in the hindpaw was seen at 48 h. At this time point, we found a significant increase in the number of nNOS‐positive cells on the ipsilateral side (Fig. 3b). Surprisingly, the difference observed using western blot analysis did not reach statistical significance, suggesting that the changes in nNOS expression occurred in a small subset of spinal neurons. Western blot analysis of a whole spinal cord quadrant would indeed not be as sensitive as immunohistochemical analysis to detect small changes. Nonetheless, this increase in nNOS expression was accompanied by a significant increase in HDAC2 S‐nitrosylation. However, when the nNOS activity was inhibited using the specific inhibitor N‐[4‐[2‐[[(3‐Chlorophenyl)methyl]amino]ethyl]phenyl]‐2‐thiophenecarboxamide dihydrochloride (ARL) 17 477 at a dose that had been shown to reduce hypersensitivity in rats (Coutinho and Gebhart 1999), there was no reduction in HDAC2 nitrosylation (Fig. S3). Establishing the time of administration of the nNOS inhibitor with respect to the time of CFA injection and tissue dissection is challenging and it is likely that we did not inhibit nNOS at the optimal time. However, other factors such as BDNF (Nott *et al*. 2008) could also be responsible for NO production and HDAC2 nitrosylation in the spinal cord after injury. Regardless of the pathway involved, an increase in HDAC2 nitrosylation following CFA injection suggests that HDAC2 inhibition of gene expression was released at least in a subset of cells. Interestingly, NO can diffuse from its site of production and cross‐membranes (Namiki *et al*. 2005), and signal even to non‐neuronal cells (Garthwaite 2008). Altogether, this implies that the site or cell specificity of HDAC2 S‐nitrosylation is hard to identify.

Overall, animal studies have demonstrated a profound pronociceptive role for NO in both inflammatory and neuropathic pain (Chu *et al*. 2005; Guan *et al*. 2007; Schmidtko *et al*. 2009) and we could hypothesize that up‐regulation of expression of pronociceptive genes following HDAC2 S‐nitrosylation might contribute to this process. For example, it had been reported that a subset of nNOS‐positive inhibitory interneurons express cFos 2 h after intraplantar formalin (Polgár *et al*. 2013). Here, looking 48 h after CFA injection in the hindpaw, the optimal time point for spinal nNOS increase, we found that 7% of nNOS‐positive neurons also expressed cFos. This observation suggests that release of cFos inhibition by HDAC2 could contribute to cFos up‐regulation following noxious stimulation. It would be an interesting advance to confirm that spinal cFos expression is, at least partly, under the control of HDAC2. A number of studies in the brain tissue and cultured cortical neurons have already shown that inhibitors of Class I HDACs can indeed potentiate cFos expression (Sng *et al*. 2005; Koppel and Timmusk 2013; Wang *et al*. 2015). However, in the absence of specific HDAC2 inhibitors, the particular role of HDAC2 in the regulation of cFos expression is more delicate to assess. Finally, we observed an up‐regulation of HDAC2 expression specifically in nNOS‐positive neurons, 48 h following CFA intraplantar injection. This offers the interesting perspective that modulation of gene expression by HDAC2 might be a process tightly regulated by a negative feedback loop.

While HDAC activity can be modulated by a number of post‐translational modifications, including nitrosylation, phosphorylation, and SUMOylation (Segré and Chiocca 2011), the level of expression of HDAC is also a key factor in HDAC‐dependent mechanisms. Indeed, HDAC2 was decreased in the hippocampus of fear‐conditioned rats after learning (Gupta‐Agarwal *et al*. 2012) and HDAC2‐knockout mice showed enhanced synaptic density and neuroplasticity (Guan *et al*. 2009). In contrast, over‐expression of HDAC2 impaired hippocampal‐dependent memory formation, decreased CA1 spine density, and impaired hippocampal long‐term potentiation in mice (Guan *et al*. 2009). Moreover, increased HDAC2 expression and decreased histone acetylation in plasticity‐related genes have been observed in memory centres in Alzheimer disease (AD) and are believed to underlie the cognitive decline observed in AD (Gräff *et al*. 2012). Interestingly, the constrain in cognitive functions resulting from HDAC2 build up is reversible, suggesting that the potential for neuronal plasticity is not lost in severely degenerated brains (Gräff *et al*. 2012). We saw no indication of global changes in spinal HDAC2 expression after peripheral injury, but did observe changes specifically in nNOS‐positive neurons and astrocytes, suggesting that the regulation of expression of HDAC2 is a highly cell‐specific process, at least at the spinal level. It is interesting to note that the presence of HDAC2 in the blood might have contaminated the HDAC2 signal in our western Blot analysis, making the exact quantification of spinal HDAC2 expression less sensitive than expected.

Our findings also suggest that HDAC2 might be important for the regulation of gene expression in astrocytes in neuropathic pain states. HDAC2 expression was indeed increased in astrocytes on the ipsilateral side, 7 days following nerve injury. Crucially, the role of astrocytes is predominant in the maintenance phase of neuropathic pain (Scholz and Woolf 2007). An increase in HDAC2 expression in astrocytes at this time point would suggest that the expression of a subset of astrocytic genes is inhibited. Interestingly, down‐regulation of the glial glutamate transporters GLAST and GLT‐1, which are both predominantly expressed in astrocytes, has previously been reported in a number of neuropathic models (Weng *et al*. 2005, 2014; Nie and Weng 2010; Zhang *et al*. 2012). This down‐regulation was shown to cause glutamate to spill into the extra synaptic space and activate postsynaptic glutamate receptors in spinal sensory neurons, leading to increased dorsal horn excitability which contributes to the development of persistent pain (Ren and Dubner 2010). The possibility that HDAC2 directly regulates astrocytic glutamate transporter expression should therefore be explored.

While our data has clearly shown that peripheral injury induces changes in HDAC2 expression and activity in the dorsal horn that might regulate injury induced changes in gene expression, the impact of HDAC2 activity on the development of injury‐induced hypersensitivity remains to be explored. Intrathecal delivery of currently available non‐specific inhibitors of HDAC and histone acetyl transferase, enzymes with opposite activity, have both been shown to temporarily improve injury‐induced mechanical hypersensitivity in subsets of pain models (Géranton and Tochiki 2015b). This clearly indicates that inhibitors that globally affect the epigenetic landscape and might regulate the expression of a large number of genes are not suitable to explore the complexity of epigenetic programmes engaged after injury and the role of individual HDAC in nociceptive signalling. The findings reported here show that future strategies to inhibit HDACs will not only have to be HDAC specific but also target different cell types independently.

## Conclusions

We have shown here that HDAC2 activity in the dorsal horn was regulated by S‐nitrosylation after injury. Considering the prominent role of NO in persistent pain states, this modification could be a key regulator of HDAC2 activity in the superficial dorsal horn. Moreover, our cell‐specific approach has revealed a complex pattern of expression for spinal HDAC2 following peripheral injury. There were no changes in global HDAC2 expression after injury, but HDAC2 was increased in nNOS‐positive neurons after CFA and in astrocytes after neuropathic injury. This indicated a cell‐specific epigenetic regulation of transcriptional programmes. Finally, it has been shown that by regulating structural and functional neuronal plasticity, not only can HDAC2 inhibit cognitive processes but also paradoxically maintain memory fidelity by preventing the modification of remote memory (Gräff *et al*. 2014). It therefore appears crucial to explore the possibility that HDAC2 could contribute to the maintenance of the undesired long‐term memory seen in chronic pain.

## Author contributions

SMG designed the experiments and wrote the manuscript. SMG, MM, OBM and KKT conducted the experiments and analysed the data. EH conducted the experiments and analysed the data presented in Figs 2, 4 and 5. KR contributed to the data presented in Figs 1 and 2. DWUO contributed to the data presented in Fig. 3, in particular the immunoprecipitation experiment. All authors read and approved the final manuscript.

## Supporting information


**Figure S1**. Negative controls for immunohistochemical double labelling of cFos and nNOS, and nNOS and HDAC2.
**Figure S2.** Rat spinal HDAC2 is mainly found in neurons and astrocytes: control using a Santa Cruz Biotechnology antibody.
**Figure S3.** Effect of nNOS inhibition on HDAC2 nitrosylation 8 h after CFA injection in the hindpaw.
