# Supplementary material for: Complex regulation of the regulator of synaptic plasticity histone deacetylase 2 in the rodent dorsal horn after peripheral injury
Source: J Neurochem. 2016 May 27;138(2):222–32. doi: 10.1111/jnc.13621 (PMC4982040; doi:10.1111/jnc.13621)
Supplement: Supplementary file 1 — Figure S1. Negative controls for immunohistochemical double labelling of cFos and nNOS, and nNOS and HDAC2. Figure S2. Rat spinal HDAC2 is mainly found in neurons and astrocytes: control using a Santa Cruz Biotechnology antibody. Figure S3. Effect of nNOS inhibition on HDAC2 nitrosylation 8 h after CFA injection in the hindpaw. [file JNC-138-222-s001.docx]

**Supplementary Material
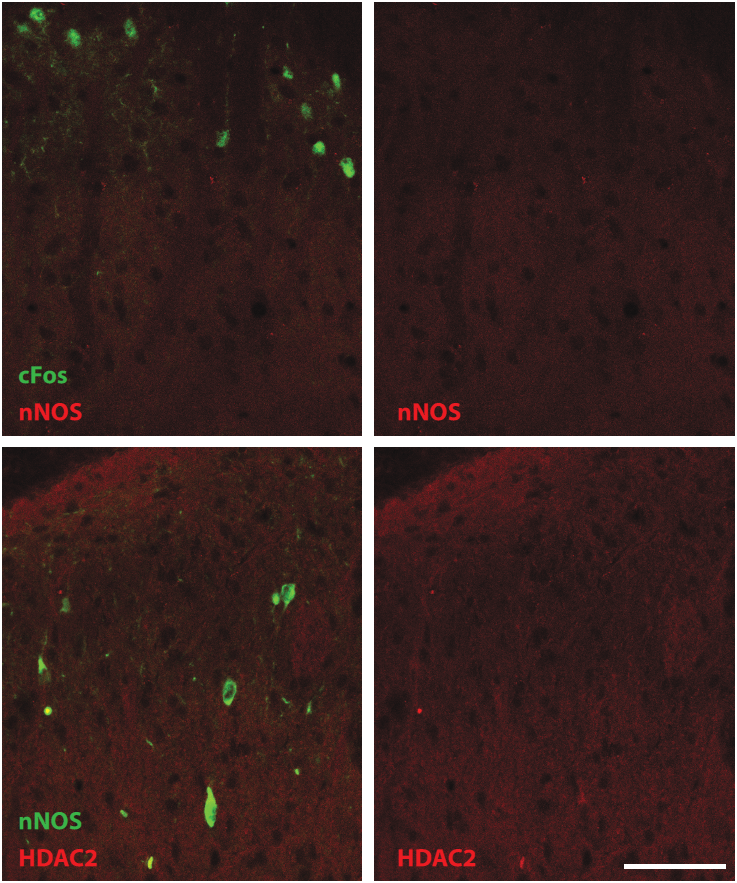
----------------------------------------------------------------------------------------------------**

**Fig.S1: Negative controls for immunohistochemical double labelling of cFos and nNOS, and nNOS and HDAC2.** cFOS and nNOS stain (green) were obtained following a TSA amplification protocol. For the HDAC2 and nNOS secondary stain (red), we omitted the primary antibody against nNOS or HDAC2 but pursued the protocol as usual for the remaining steps (see Methods). There were no red signal coming from the cFOS and nNOS positive signals indicating that co-expression in the same cells of cFos and nNOS, as well as nNOS and HDAC2, presented in Figure 3 is not due to cross reactivity of the secondary antibodies. Spinal cord sections were obtained from rats injected with CFA 2h before tissue dissection. Scale bar: 50µm.


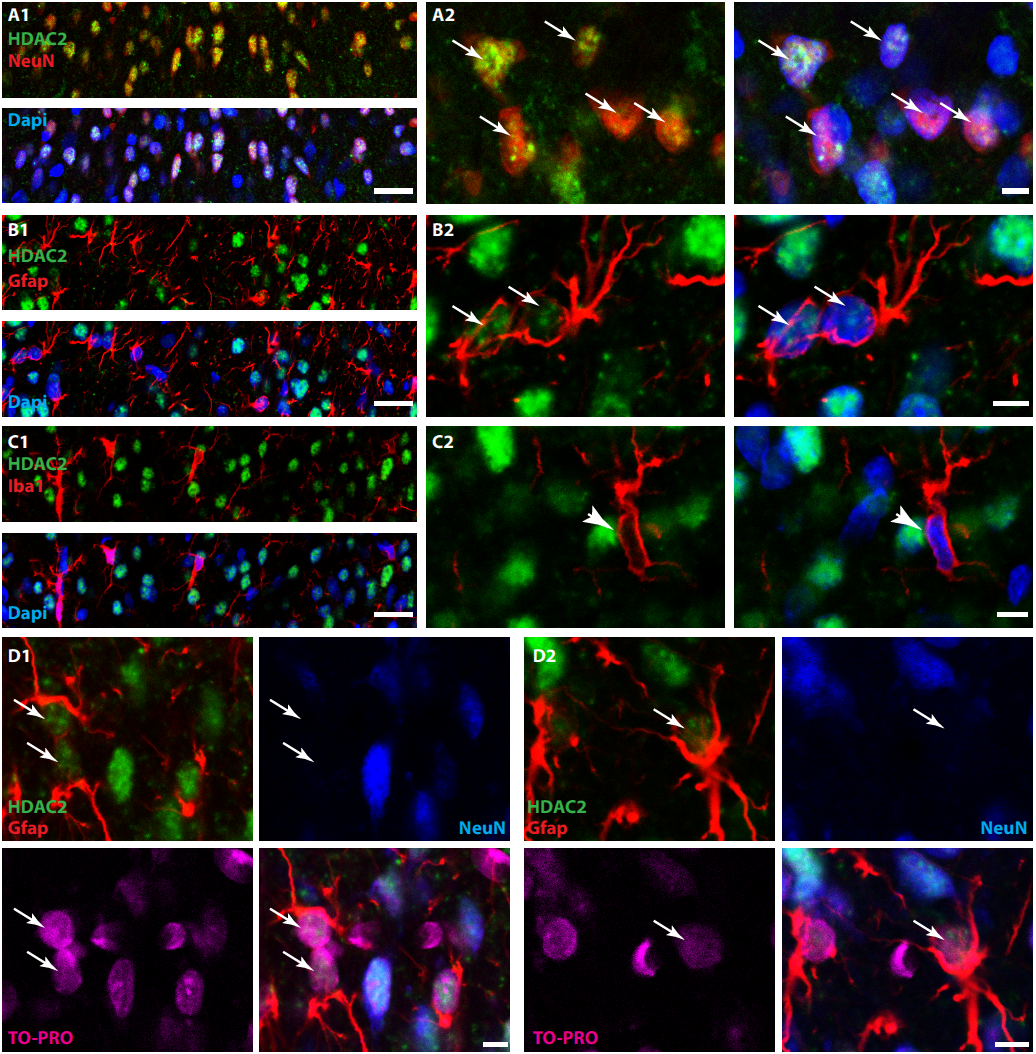


**Figure S2: Rat spinal HDAC2 is mainly found in neurones and astrocytes: control using a Santa Cruz Biotechnology antibody**. HDAC2 expression in the rat superficial dorsal horn was investigated using immunohistochemistry. **A1-2**/ Expression of HDAC2 (green) in dorsal horn neurones (labelled with NeuN, red). Dapi (blue stain) was used to label nuclei. Coexistence is seen in yellow. All neurones were expressing HDAC2. **B1-2**/ Expression of HDAC2 (green) in dorsal horn astrocytes (labelled with Gfap, red). Since Gfap does not stain the astrocytic nucleus, there is no overlap between the 2 stains. Dapi (blue stain) was used to label nuclei. **C1-2**/ Expression of HDAC2 (green) in dorsal horn microglia labelled with Iba1 (red). Dapi (blue stain) was used to label nuclei. There was no obvious expression of HDAC2 in microglia. **D1-2**/ Expression of HDAC2 (green) in dorsal horn neurones (blue) and astrocytes (red). TO-PRO (cyan stain) was used to label nuclei. Scale bar: A1, B1, C1: 20 µm; A2, B2, C2, D1, D2: 5 µm. Arrows in A point at HDAC2 expressed in neuronal nuclei. Arrows in B and D point at HDAC2 expressed in astrocytic nuclei. Arrow head in C points at the absence of HDAC2 stain in microglial nuclei.


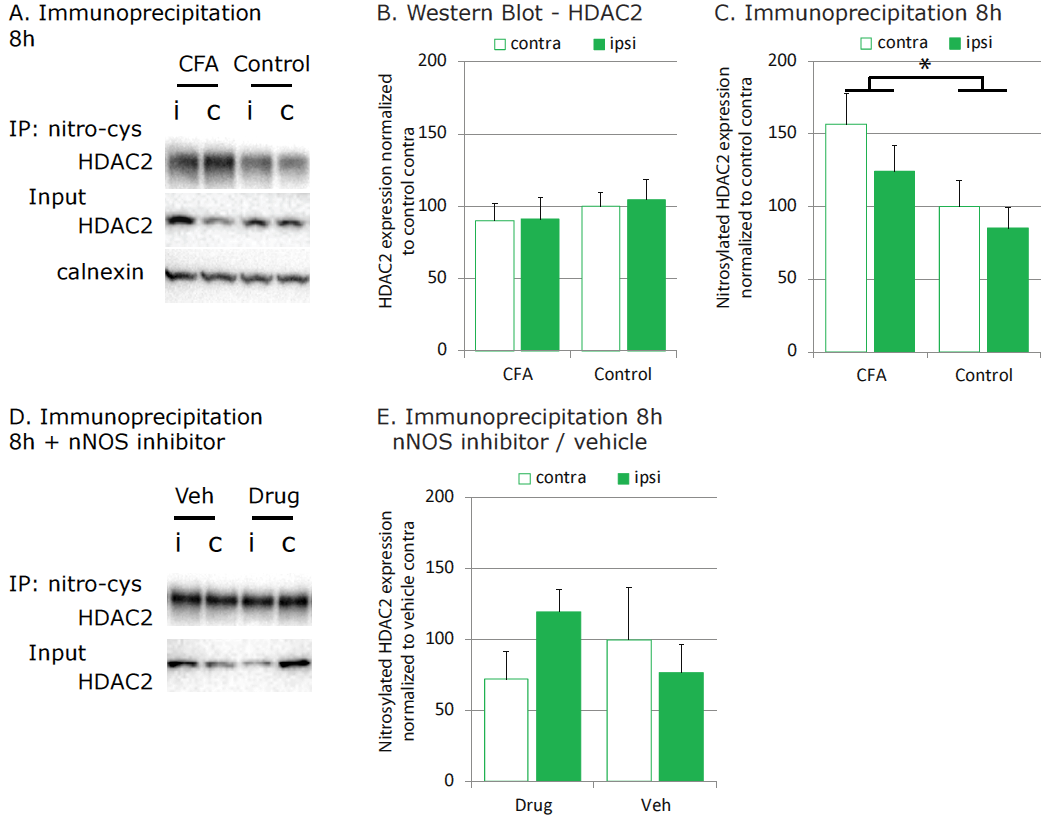


**Figure S3: Effect of nNOS inhibition on HDAC2 nitrosylation 8h after CFA injection in the hindpaw.** **A**/ Representative blots after immunoprecipitation with nitro-cysteine antibody. Tissue was collected 8h post CFA injection in the hindpaw or control treatment. **B**/ Western blot analysis of HDAC2 expression indicated no changes 8h following intraplantar CFA. Data normalized to contra control (100%). N=5/5. **C**/ Quantification of immunoprecipitation blots as shown in A. There was a significant effect of CFA injection on HDAC2 nitrosylation. ANOVA TREAT: F_(1,8)_= 5.5, P<0.05. **D**/ Representative blots after immunoprecipitation with nitro-cysteine antibody. Tissue was collected 8h post CFA injection in the hindpaw and 3h post intrathecal injection of the nNOS inhibitor ARL 17477 dihydrochloride (TOCRIS), at a dose of 50mM in 10µl (500nmol; intrathecal delivery), or vehicle (water); N=5/5. **E**/ Quantification of immunoprecipitation blots as shown in D. There was no effect of ARL 17477 administration as described in D on HDAC2 nitrosylation levels 8h after CFA injection in the hindpaw. i: ipsi; c: contra; Drug: ARL 17477; Veh: water; IP: immunoprecipitation.
